# Supplementary material for: Multiband topological heterojunctions on the surface nanoscale axial photonics platform
Source: Nat Commun. 2026 Sep 29;17:10071. doi: 10.1038/s41467-026-76041-1 (PMC13623952; doi:10.1038/s41467-026-76041-1)
Supplement: Supplementary file 1 [file 41467_2026_76041_MOESM1_ESM.pdf]

Supplementary Information for "Multiband Topological Heterojunctions on the Surface Nanoscale Axial Photonics Platform"

Nathaniel Fried[1,2], Dashiell L. P. Vitullo[2*], Avik Dutt[1,3,4*]

[1]Institute for Physical Science and Technology, University of Maryland, 4254 Stadium Drive, College Park, 20742, MD, USA.
[2]DEVCOM Army Research Laboratory, 2800 Powder Mill Rd, Adelphi, 20783, MD, USA.
[3]National Quantum Laboratory (Qlab), University of Maryland, 4505 Campus Drive, College Park, 20740, MD, USA.
[4]Department of Mechanical Engineering, University of Maryland, 4298 Campus Dr, College Park, 20742, MD, USA.

*Corresponding author(s). E-mail(s): dashiell.l.vitullo.civ@army.mil; avikdutt@umd.edu;
Contributing authors: friednr@umd.edu;

1 Supplementary Note 1: Fiber Tapering

A Vytran GPX-3800 Glass Processing System system was used to taper SMF-28 fibers to a diameter of approximately 1 μm to ensure only a single mode could be supported. The tapering processes was broken into two steps: initially a 2 mm section of the fiber was heated and then stretched apart by 9 mm on each side, followed by heating a 4 mm region of the fiber and stretching by 8 mm on each side, resulting in an increase in the fiber length by 34 mm.

2 Supplementary Note 2: SNAP Spectrum Fitting & Modeling

Fig. 1 shows a measured and model spectrogram of a lone SNAP resonator. The model is obtained by assuming a super-Gaussian ERV curve:

$$\Delta r(z) = \frac{\bar{r}}{\bar{\lambda}}\left[\Lambda \exp\left(-\left(\frac{z-z_0}{\sqrt{2}\sigma}\right)^{2p}\right) + h\right], \tag{1}$$

where Λ represents the height of the resonator along the wavelength axis, z_0 its central location, σ its width, and p is a factor representing the "sharpness" of the ERV curve in comparison to a normal Gaussian curve. $\bar{r}$ and $\bar{\lambda}$ are defined as before, and h accounts for errors in the estimate of $\bar{\lambda}$. These parameters are fit by finding the bare resonant wavelengths $\Delta\lambda_q^{\mathrm{meas}}$ by locating the transmission minimum within the spectrogram (specifically at their caustics to avoid the effects of the taper renormalization [1]), then determining the eigenvalues of Eq. 6 from this potential corresponding to the bound modes $\Delta\lambda_q^{\mathrm{model}}$. Then a gradient-descent minimization of the listed parameters with a cost function equal to sum of the squared differences of $\Delta\lambda_q^{\mathrm{meas}}$ and $\Delta\lambda_q^{\mathrm{model}}$ for $q \in [1, 10]$ can be employed. In Supplementary Fig. 1(b), the result is that $\Lambda = 1.240$ nm, $\sigma = 39.7$ μm, and $p = 1.117$.

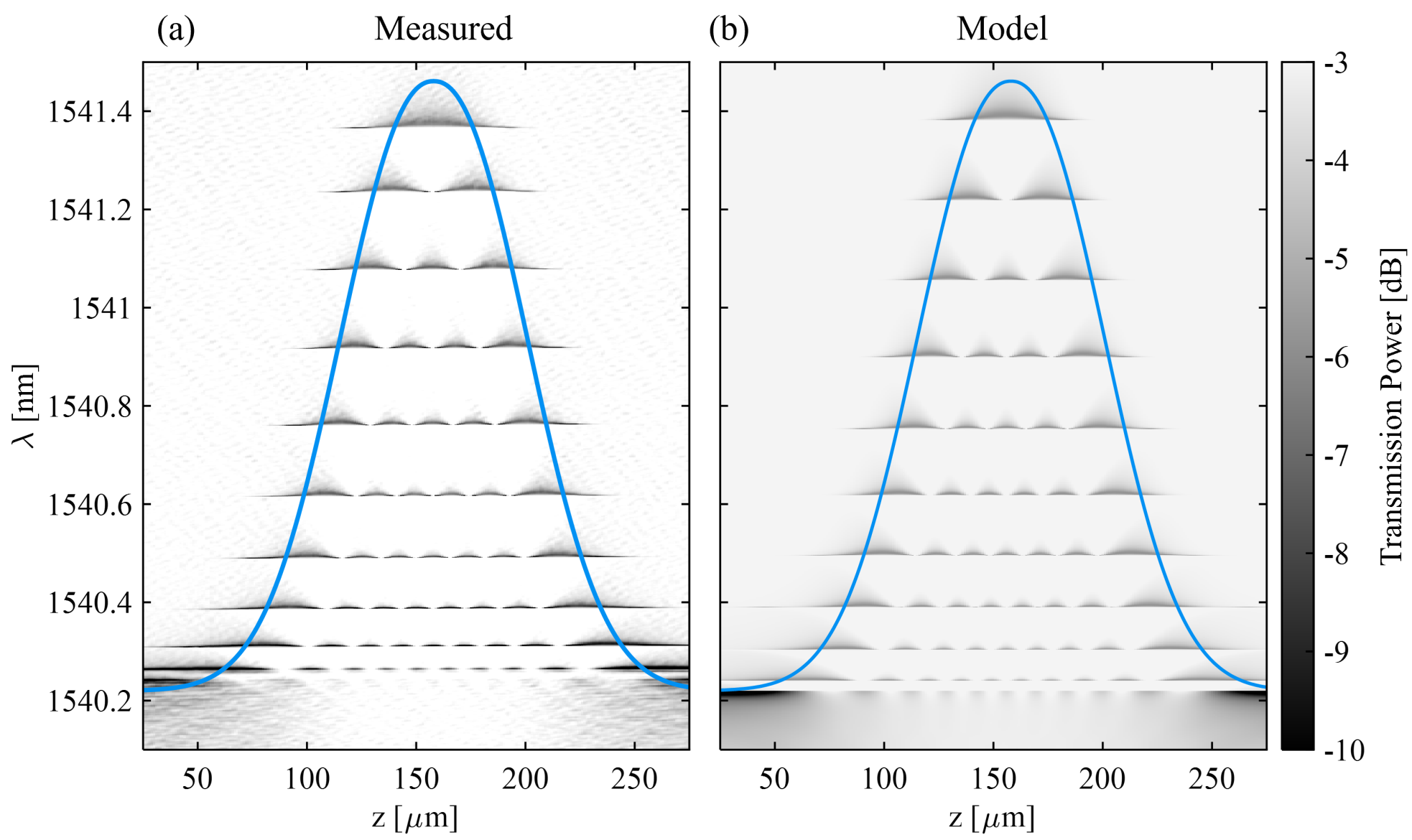

Supplementary Fig. 1: **(a)** Measured and **(b)** Fitted Model Spectrogram of a SNAP resonator comparable to those created in this work.

Resonator #	1	2	3	4	5
Λ (nm)	1.108	1.119	1.106	1.093	1.124
σ (μm)	37.2	39.6	35.8	37.0	38.7

Table 2: Fitted values of parameters for each of the resonators in Fig. 2. Every resonator had a common value of p fitted, for which the result was $p = 1.144$.

When multiple SNAP resonators labeled by position index j, each individually corresponding to a super-Gaussian ERV profile $\Delta r_j(z)$, are fabricated adjacent to each other, the modes of the overall structure are determined by some cumulative ERV profile $\Delta r(z)$. Due to the effects of the nonlinearity of the CO_2 annealing process on positions that receive multiple contributions, it will not generally be the case that multiple identical laser pulse exposures at nearby axial positions will give rise to a net ERV profile equal to the sum of individual super-Gaussian ERV curves. This means we find the cumulative ERV at any location more closely resembles the *maximum* of the individual ERV profiles at any given location. The ERV profiles are better modeled as "overwriting" each other,

$$\Delta r(z) \approx \max_j \Delta r_j(z), \tag{2}$$

as opposed to

$$\Delta r(z) = \sum_j \Delta r_j(z). \tag{3}$$

Supplementary Fig. 2 compares ERV profile models given by Eq. (2) and Eq. (3) by applying them to the measured 5-site SSH2 lattice (analyzed in Fig. 2). In each diagram, the σ and p parameters are taken from the results of fitting the lone resonator in Supplementary Fig. 1, but Λ has been rescaled to better represent the size of these resonators (i.e. they are reduced from 1.24 nm to the values shown in Table 2). The ERV curve shown in Supplementary Figs. 2(a) and 2(b) are computed under the overwriting model (Eq. (2)), whereas the ERV curve of (c) and (d) are computed under the linear model (Eq. (3)). The ERV profile using the overwriting profile gives rise to a modal spectrum that is a much better match to the measured data than the linear model. In this situation, it is clear the overwriting assumption is both the superior model and an accurate one, motivating its use in fitting for Fig. 3. The ERV profile for each resonator was permitted to have a distinct width σ and Λ, but a common p value. The results are $p = 1.144$ and the values for Λ and σ are written in Table 2. The similarity between these fitted widths and the p value to that of the individual resonator in Supplementary Fig. 1 further affirms the validity the of the ERV-overwriting assumption.

It should be noted that the overwriting model underestimates ERV growth in situations where the intersite spacing Δz is comparable to the mode widths Δw_q (and/or the resonator widths σ). This can be easily verified by recognizing that the

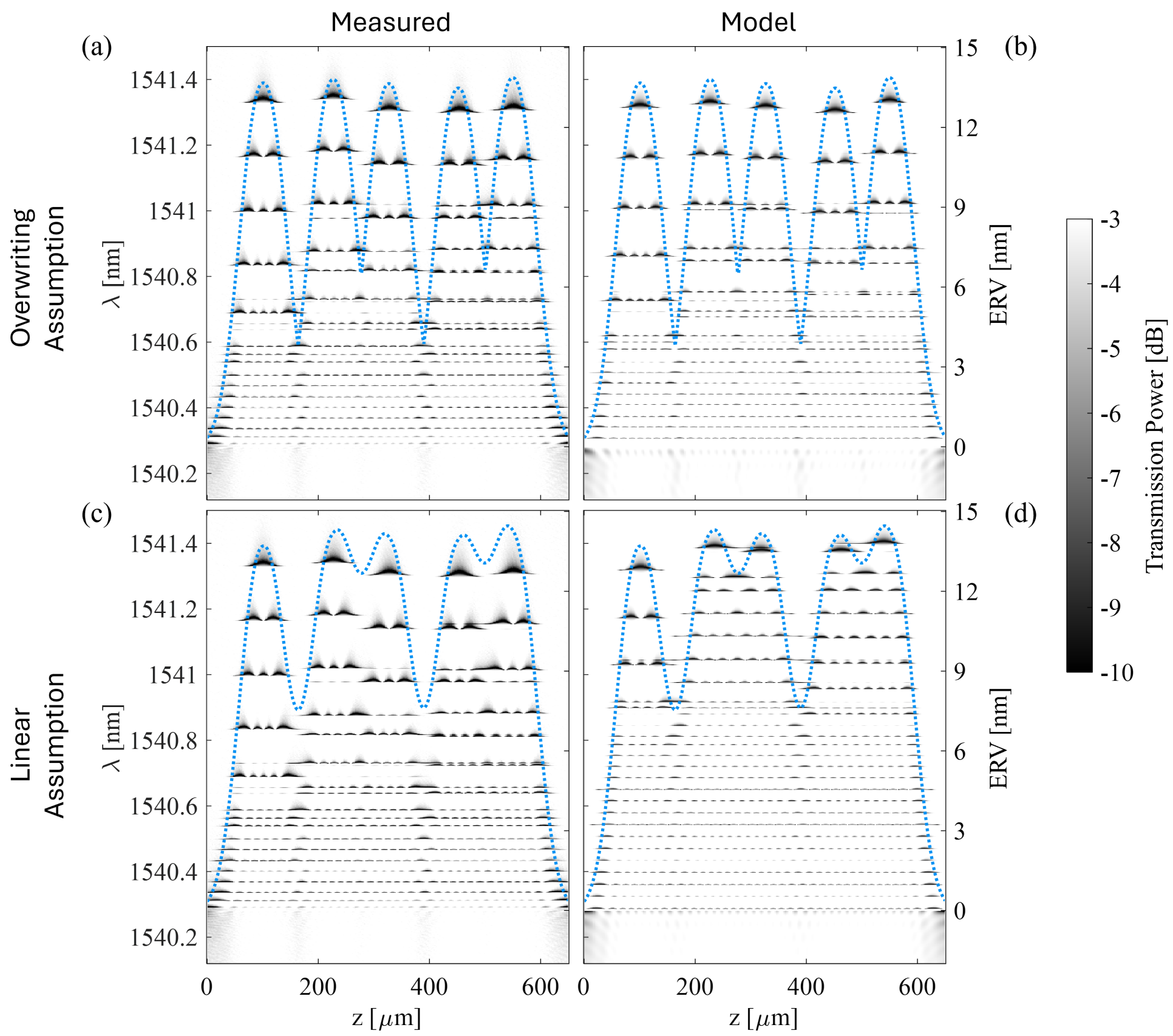

Supplementary Fig. 2: Comparison of modeling approaches for 5-site SSH model shown in Fig. 3. Measured spectrograms of SNAP device from Fig. 2 (a,c) of the main manuscript where each resonator has an ERV curve based on fit parameters of Supplementary Fig. 1 but with $\Lambda = 1.11$ nm and different assumptions about how ERV from separate exposures combine. In (a) it is assumed that the ERV of the device abides by eq. (2) whereas (c) assumes ERV growth abides by eq. (3). The corresponding computed spectrograms with the same ERV curves are depicted in (b) and (d) respectively. The ERV curve in (c) fails to follow the edges of the mode profiles, and spectrogram (d) does not resemble the measured ones in (a) and (c) as a whole, indicating a failure of the linear assumption of ERV growth.

overwriting model implies there would be no ERV change at all during the post-processing steps in which the resonances are matched.

3 Supplementary Note 3: SSH2/SSH4 Heterojunction Comparisons

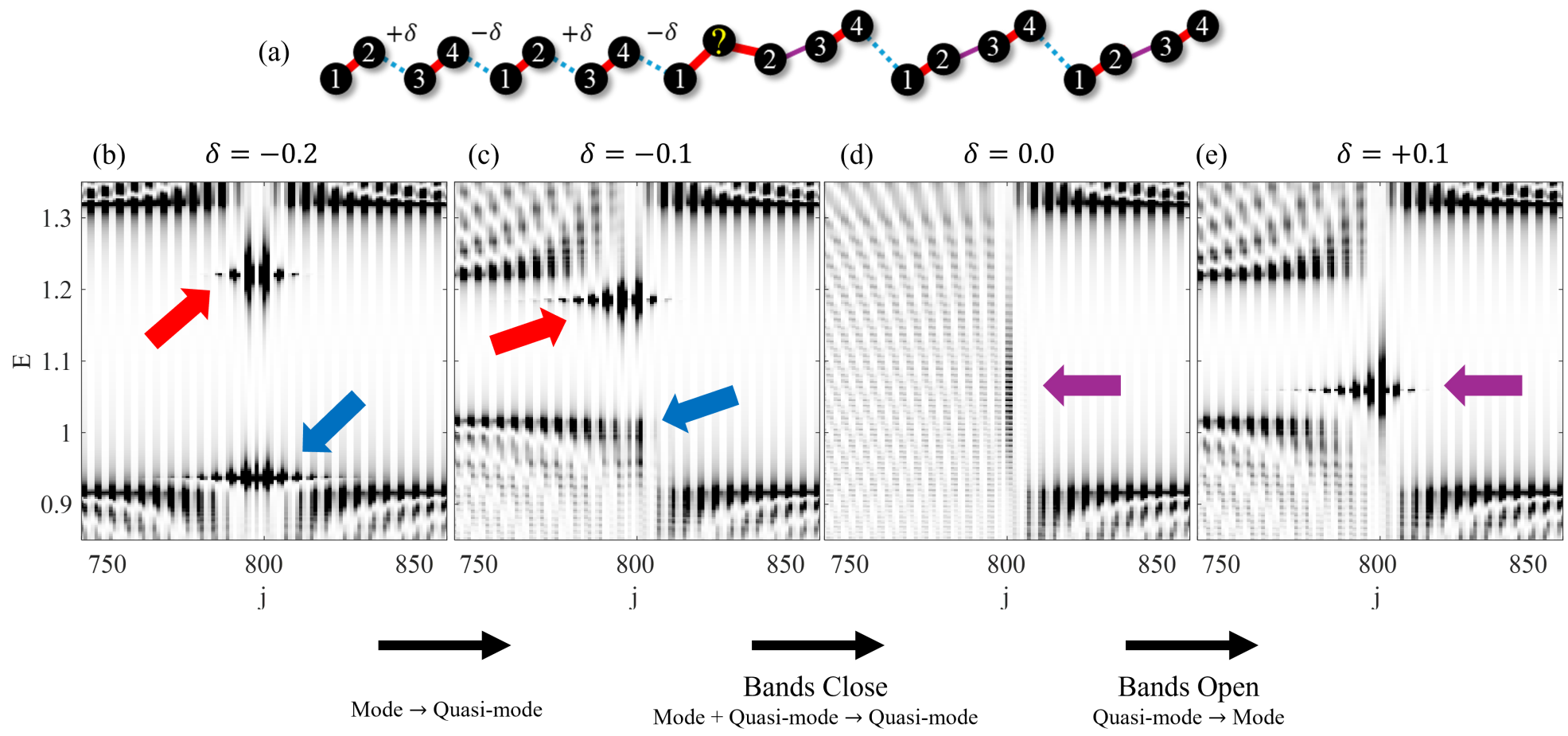

Supplementary Fig. 3: **(a)** Polymer model of the perturbed lattice heterojunction, and (b-e) partial views of the simulated bare tight-binding Green's functions (arb. units for intensity) showing how the bare mode structure varies with δ across the critical point at $\delta = 0$. **(b)** $\delta = -0.2$ Two real modes (red and blue arrows) are visible within the gap. **(c)** $\delta = -0.1$ The lower of the two modes (blue arrow) from is converted into a quasi-mode as it enters the band. **(d)** $\delta = 0$ The closing of the bandgap cause the quasi-mode and real mode to merge into a single quasi-mode (purple arrow). **(e)** $\delta = +0.1$ The quasi-mode becomes a real mode (purple arrow) after the bandgap opens again.

Supplementary Fig. 3 contains close-up views of the auxiliary bandgaps of heterojunctions like that of Fig. 3, but with the δ perturbations as described in the discussion section, such that the couplings are $v_L = [1, 0.5 + \delta, 1, 0.5 - \delta]$ with $N_L = 200$ on the left (formerly SSH2) side, and $v_R = [1, 0.7, 1, 0.3]$ with $N_R = 200$ on the right side (Supplementary Fig. 3(a)). A nonzero δ opens a bandgap in the middle of each of the SSH2 bands, placing it on similar footing with SSH4. For all values of δ there is a zero-energy mode and the pair of modes above and below all the bands like in Fig. 3(b-c), which is why only the auxiliary bandgap is shown. For $\delta = -0.2$ (when the bandgaps of the left side and right side are equal, and the system has exact inversion symmetry about the interface), (Supplementary Fig. 3(b)) there are two bound modes (red and blue arrows) present in the auxiliary band gap. However, (Supplementary Fig. 3(c)) for $\delta < 0$ except $\delta \neq -0.2$, the lower (blue) of the two modes becomes a quasi-mode, as its energy falls within the bandgap (For $\delta < -0.2$, the spectrograms are formally equivalent to Supplementary Fig. 3(c) but with the left and right sides switched). When δ reaches zero as the auxiliary bandgaps close (Supplementary Fig.

3(d)), the quasi-mode and real mode combine into a single quasi-mode (purple arrow). Past (Supplementary Fig. 3(e)) $\delta > 0$, there is only a single bound mode within the auxiliary bandgap, implying that $\delta = 0$ is a critical point of the system.

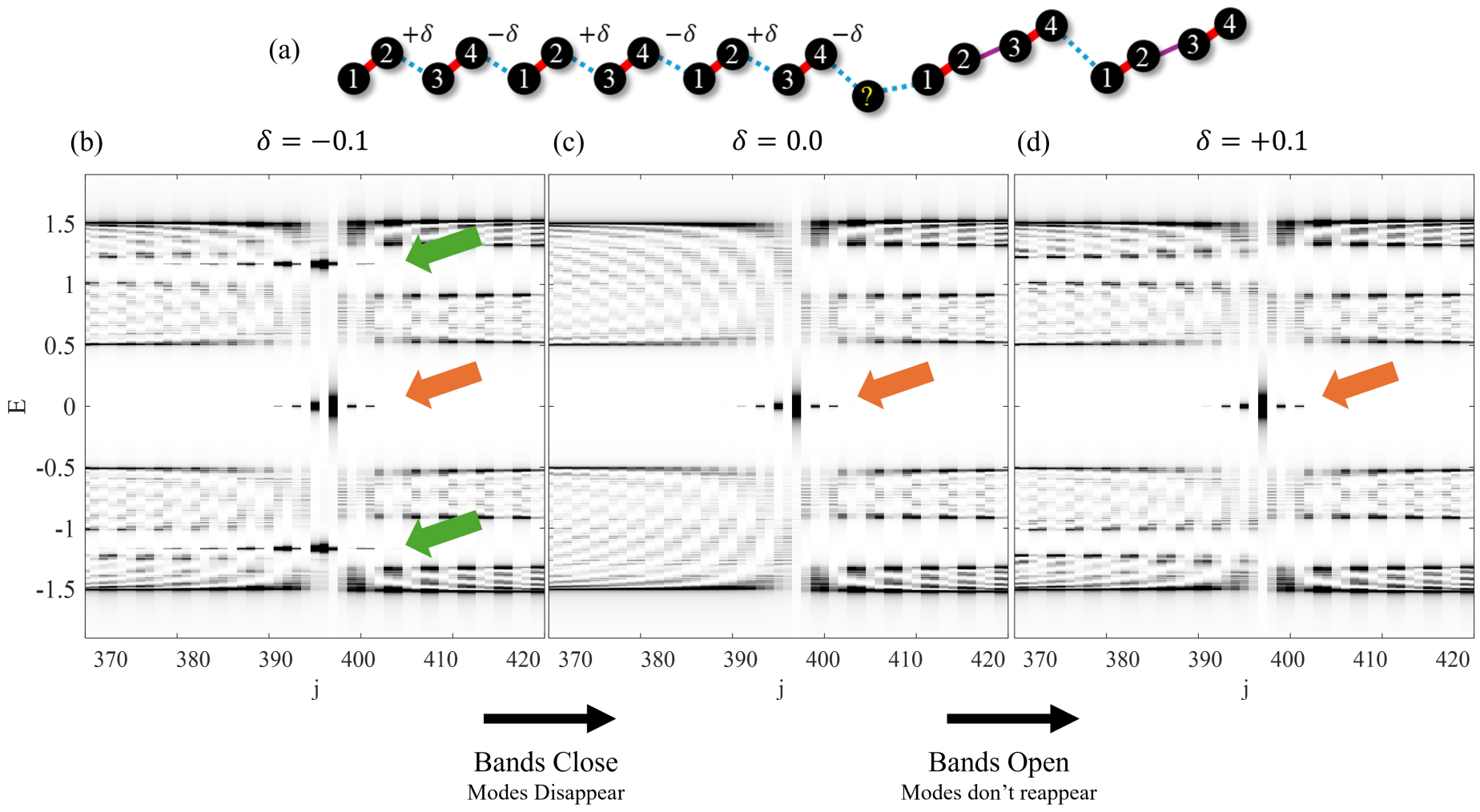

Supplementary Fig. 4: **(a)** Locally deformed and perturbed lattice. (b-d) bare tight-binding Green's functions of systems that have been perturbed by δ, but have also been deformed to possess the minimum number of interface modes. **(b)** For $\delta < 0$, there are three modes- a zero energy bound mode (orange), and two modes within the auxiliary bandgaps (green). **(c)** For $\delta = 0$, the auxiliary bound modes disappear, and don't reappear for (d) $\delta > 0$.

We also examine the δ-perturbed heterojunction after topology-preserving local deformations (e.g. Supplementary Fig. 4(a)) to find the minimum number of interface modes – a topologically robust quantity by definition with which we can compare the number of apparent modes (Supplementary Fig. 3) and the sublattice displacements. Notably, the zero-energy mode continues to appear in every case (Supplementary Fig. 4(b-d)), and quasi-modes fail to appear in any case. At the $\delta = 0$ (Supplementary Fig. 4(c)) band closing point, the interface transitions from hosting 3 bound modes ($\delta < 0$, Supplementary Fig. 4(b)) to having only a single bound mode (Supplementary Fig. 4(d)), re-affirming $\delta = 0$ as a critical point.

We list the real and quasi-modes for each case depicted in Supplementary Fig. 3 in Table 3, along with their sum mod 4 (the "total" mode count) and the sublattice displacements from Table 1. The total net mode count is equal to 3 for $\delta < 0$ and 1 otherwise, suggesting that $\delta = 0$ is a critical point. For all values of δ, the minimum mode count matches the total mode rather than the real mode count (mod 4), suggesting that quasi-modes are a valid indicator of topological phase changes just like real modes (i.e., quasi-modes are a form of topological "charge"). Likewise, the sublattice displacements almost always line up with the net and minimum mode count, with the

critical point $\delta = 0$ as the only exception. The correct result can be obtained by recognizing that the predicted number of modes should only be unique mod 2 for $\delta = 0$ (since the left side has two bands), or by taking the minimum of all the predictions across the sublattice.

Mode Count	$\delta = -0.2$	$\delta \neq -0.2$ & $\delta < 0$	$\delta = 0$	$0 < \delta$
Real	7	5	3	5
Quasi-	0	2	2	0
Total	3	3	1	1
$D_L - D_R$	3	3	3 (1)	1
Minimum	3	3	1	1

Table 3: Tabulation of modes and quasi-modes observed in spectrograms from Supplementary Fig. 3(b-d), with the total modes (sum of the modes and quasi-modes mod 4). Minimum modes consider the lowest number of bound modes localized to the interface under local deformations like in Supplementary Fig. 4. Additionally, the minimum of the changes in the sublattice displacement across the sublattice from Table 1 are provided. In all cases, the net modes coincide with the minimum modes and the minimum of the displacement across the sublattice. The $\delta = 0$ point is a critical point between the system having 1 or 3 net topological modes.

4 Supplementary Note 4: Distribution of Boundary Modes

Equation 4 can be refined further to determine the specific number of bound modes on either side of the lattice. Specifically, we can consider it to be a function of the beginning and ending sites of the lattice $Q(s_L, s_1)$, and that it is the sum of the sum of the bound modes on the positive $Q_+(s_L)$ and negative $Q_-(s_1)$ end of the lattice, each functions of their corresponding ending site and the bulk topology:

$$Q(s_L, s_1) = Q_+(s_L) + Q_-(s_1) = \big[D(s_{L+1}) - 1\big] - \big[D(s_1) - \ell\big]. \tag{4}$$

This form of Eq. 4 suggests we can guess that the one-sided bound modes should be able to be expressed in terms of the sublattice displacement of the corresponding side. We write the equations in terms of the "relative" sublattice displacement $d(s) = D(s) - s$

$$+Q_+(s_L) = [s_{L+1} - 1] + d(s_{L+1}) + C, \tag{5}$$
$$-Q_-(s_1) = [s_0 \quad - \ell] + d(s_0 \quad) + C, \tag{6}$$

for some constant C, which must be topology-dependent but not boundary-dependent. It can be ascribed meaning in terms of the average imbalance of bound states across the lattice $Q_+ - Q_-$ over all possible terminations of the boundary (all values of s_L and s_1):

$$\Delta Q = \frac{1}{2\ell^2} \sum_{s'=1}^{\ell} \sum_{s=1}^{\ell} \Big[Q_+(s') - Q_-(s) \Big] = C + \bar{d}, \tag{7}$$

where $\bar{d} = \frac{1}{\ell} \sum_{s=1}^{\ell} d(s)$ is the "mean" relative sublattice displacement. Eq. (7) thus serves as a definition of another new topological invariant. In terms of ΔQ, we can write more meaningful equations for the numbers of boundary modes on each end of the lattice:

$$+Q_+(s_L) = [s_{L+1} - 1] + \big[d(s_{L+1}) - \bar{d}\big] + \Delta Q, \tag{8}$$

$$-Q_-(s_1) = [s_0 \quad - \ell] + \ \big[d(s_0 \quad) - \bar{d}\big] + \Delta Q. \tag{9}$$

Particularly, in the case that the lattice has inversion symmetry [2, 3], we can immediately conclude that $\Delta Q = 0$, making the one-sided boundary mode counts correspond directly the sublattice displacements. Without inversion symmetry, ΔQ must be inferred by determining the number of bound states directly. In such scenarios, one would be able to predict

Eq. (8) can also be applied to analyzing interfaces between inversion-symmetric lattices by considering the number of interface states Q_I to be the difference in number of bound modes each side of the lattice "contributes" to the interface modulo the number possible topological phases of the interface.

$$Q_{\text{Int}} = (Q_{+,\text{Left}} - Q_{-,\text{Right}}) \mod \ell_{\text{phases}}. \tag{10}$$

For example, considering the structure in Fig. 3 to be a 10 site SSH2 lattice attached to an 11 site SSH4 lattice, we will get $Q_{\text{Int}} = (0 - 3) \mod 2 = 1$. Taken the other way (11 site SSH2 lattice attached to a 10 site SSH4 lattice), we get the same answer of $Q_{\text{Int}} = (1 - 0) \mod 2 = 1$.
