# Supplementary material for: Multiband topological heterojunctions on the surface nanoscale axial photonics platform
Source: Nat Commun. 2026 Sep 29;17:10071. doi: 10.1038/s41467-026-76041-1 (PMC13623952; doi:10.1038/s41467-026-76041-1)
Supplement: Supplementary file 2 [file 41467_2026_76041_MOESM2_ESM.pdf]

nature portfolio

Peer Review File

Multiband Topological Heterojunctions on the Surface Nanoscale Axial Photonics Platform

Corresponding Author: Professor Avik Dutt

Parts of this Peer Review File have been redacted as indicated to remove third-party material.

This file contains all reviewer reports in order by version, followed by all author rebuttals in order by version.

Version 0:

Reviewer comments:

Reviewer #1

(Remarks to the Author)
This manuscript reports an impressive and timely experimental demonstration of multiband topological heterojunctions implemented on the SNAP platform, combining extraordinary fabrication precision with elegant topological photonics concepts. The work convincingly establishes SNAP as a uniquely powerful platform for analogue Hamiltonian simulation, especially in regimes that require sub-angstrom resonance matching across large resonator arrays, which is extremely challenging for integrated photonics.

Overall, the manuscript is well written, and of clear interest to the broad communities working on topological photonics, analogue simulation, and microresonator physics. The fabrication precision, spectral resolution, and level of experimental control are outstanding. I believe this work is very well suited for publication in Nature Communications after minor revisions.

Comments
1.The statement that this approach is a “reversal of the tight-binding approximation” is interesting but could be made clearer. A short intuitive sentence explaining this statement would improve accessibility.
2.When reporting coupling strengths as wavelength splittings of several picometers, it may be helpful to translate these into frequency units (GHz) at least once, to give readers a better physical sense of the interaction scale.
3.A definition of quasi-modes when they are first mentioned would improve readability, especially for non-experts in Green’s-function spectroscopy.
4.A short intuitive explanation that band touching prevents assigning a unique bulk invariant would help readers connect this to standard topological phase-transition concepts.
5.The CO_2 laser beam drawn in Fig. 1(a) looks more like a parallel than a focused beam. Since the fabrication relies on strong spatial localization of heating, it would be helpful to redraw the beam with a visible waist at the fiber surface. As it stands, the image may mislead readers unfamiliar with the SNAP fabrication process.
6.Occasional misprints appear, e.g., “except except” in Sec. 4 and “is is clear” in Appendix B.

Reviewer #2

(Remarks to the Author)
This manuscript presents a well-executed experimental and theoretical study on the realization of topological photonic lattices using the SNAP platform. The work demonstrates the experimental implementation of SSH models and their multiband heterojunctions in SNAP devices, supported by rigorous theoretical analysis. The results are novel, the methodology is sound, and the findings are of potential interest to the broader communities in topological photonics.

1. While the manuscript cites relevant literature, a more explicit comparison with other platforms (e.g., integrated photonic circuits, superconducting qubits) in terms of performance metrics (Q-factor, scalability, tuning precision) would strengthen the case for SNAP’s advantages. The claim that this is the "first heterojunction of higher-order SSH lattices" should be supported by a brief discussion of why such structures have not been realized elsewhere, if possible.

2. The manuscript briefly mentions nonlinear effects and synthetic dimensions as future directions but does not critically discuss the current limitations of the SNAP platform (e.g., maximum lattice size, sensitivity to environmental noise, scalability beyond 1D). A dedicated paragraph on challenges and prospects would provide a more balanced perspective.

3. The development of a generalized topological polarization method for multiband heterojunctions is a notable theoretical contribution. However, the connection between Eq. (6)-(7) and physical observables could be elaborated. A more intuitive explanation of the "sublattice displacement" and its relationship to measurable quantities would strengthen the theory section.

4. The extension of the method to higher dimensions is suggested but not elaborated. A brief speculative discussion on how SNAP could emulate 2D/3D topological models would highlight the platform's future potential.

5. The authors discussed various Hamiltonian simulation platforms; however, many of the cited references are rather dated. It is recommended that the authors include more recent publications to better reflect the current progress in the field. For instance, Science Bulletin Volume 70, Issue 8, 30 April 2025, Pages 1347-1360; Nature Reviews Physics, Volume 7, pages 606–620 (2025); Photonics Insights, Vol. 4, Issue 2, R06 (June 2025).

Reviewer #3

(Remarks to the Author)
This manuscript investigates the structure of axial modes in a system of coupled SNAP microresonators. Using a well-known technique of introducing an effective radius variation into a standard optical fiber by heating it with a CO2 laser (10.1364/OE.20.027896, 10.1364/OE.20.010684), a system of coupled resonators was implemented that forms the Su-Schrieffer-Heeger (SSH) lattice. The approximation of the measured transmission spectra of the fabricated system was performed using the theory developed by Mikhail Sumetsky (10.1364/OE.19.026470, 10.1364/OE.20.022537), in analogy with how this was done for other systems of coupled SNAP resonators (10.1364/oe.21.015268, 10.1364/ol.433077). The results of the work show the presence in such a system of both edge topological modes and axial modes arising at the junction of changes in the lattice period.
The authors argue that the SNAP platform is the most suitable for analog Hamiltonian simulation, which is undeniable, because the axial part of the wave equation, which describes the field distributions in a cylindrical resonator with a radius variation, coincides in its form with the one-dimensional stationary Schrödinger equation. This also opens the possibility to study topological modes in 1D lattices.
However, the SNAP axial modes have a critical distinction from those in other topological photonic systems. In various types of topological photonic systems, the study of which has gained popularity recently, the presence of topological modes in the system leads to the emergence of non-trivial linear and nonlinear dynamics (10.1038/s41467-018-05461-5, 10.1038/s41567-021-01492-w, 10.1038/s41467-025-59941-6, 10.1038/NPHOTON.2014.248, 10.1038/s41467-023-40238-5, 10.1038/s41566-017-0048-5, 10.1038/nphoton.2013.274). In the SNAP system, the axial modes are stationary along the microresonator axis, and the field dynamics lie in the perpendicular plane. This makes it unclear what non-trivial dynamics can be expected in this system. Topological axial modes and axial modes numbered q=1-3 (Fig. 1) are equally isolated from other axial modes, and from the perspective of the linear and nonlinear dynamics that can develop in the fiber cross-section, it seems that it makes no difference which mode we pump with the radiation source. In both cases, we will have a stationary field along the microresonator axis, and the topological property of the axial distribution, due to the smallness of the effective radius variation, cannot affect the spatial distribution and dynamics of the field in the cross-section (this is the fundamental assumption of the theory when we proceed to separate the variables of the wave equation).
For example, in systems of coupled ring microresonators, nontrivial dynamics and topological soliton solutions are observed because the radiation propagates in the plane of topological modes (10.1126/science.ado0053, 10.1038/s42005-023-01438-z). A SNAP microresonator is analogous to a system of coupled ring microresonators arranged in a row and placed edgewise. Clearly, in this case, the field dynamics along the perimeter of the rings will coincide with the dynamics in a single ring.
Furthermore, the claim that the theory can be generalized to a two-dimensional lattice also seems non-obvious and was not explained in the text of the article. The linear coupling between modes with different polarizations and radial numbers is not experimentally controlled and arises due to Rayleigh scattering by fiber defects. For such coupling to be effective, the resonant wavelengths of axial series with different radial numbers or with different polarizations must overlap. Such overlap is possible, but it will lead to a linear coupling between modes with different axial numbers in the series. It is not entirely clear how such a coupling can be used for scaling.
Though the present work demonstrates a new configuration of the SNAP platform with topological properties of axial modes, it cannot make a broad impact because non-trivial light dynamics are associated with azimuthal modes only. Therefore, I recommend publishing this manuscript elsewhere in a more specialized journal.

Reviewer #4

(Remarks to the Author)
The authors report the implementation of an optical analog of a Hamiltonian system: the Su-Schrieffer-Heeger (SSH2) model and one generalization (SSH4). The experimental implementation is made with surface nanoscale axial photonics (SNAP) optical microresonators, of a simple 5-unit SSH2 chain, and a longer chain with an interface between SSH2 and SSH4 lattices. The authors then introduce theory to explain what happens at the interface of an SSH2 and SSH4, since that topic has been less explored.

Overall the manuscript is well written, very clear, and all the claims are supported with credible evidence. The topic is very timely, as optical implementations of topological systems are being actively explored and, as far to our current knowledge, there are no other topological systems implemented in SNAP resonators.

The analysis of the coupling as a function of the distance between resonators was particularly interesting.

There no major issues in the manuscript to point out, but rather a set of minor points that could potentially be clarified. These are mostly in the experimental part, due to a lack of theoretical depth to find out serious issues in the theoretical discussion (which looks fine to a lightly trained eye).

1) According to the fabrication description, the post-processing was focused on optimizing the q=3 axial mode lattice (lines 312-313). Then, the analysis is centered on the axial mode lattices with q=4,5 (with good reason, as those show very clear effects). When looking at Figure 3a, it seems that the q=3 mode does have some hint of coupling showing some splitting of states. A bit of analysis on what is going on in that case would be a good addition (ie are the couplings low enough that there is no possibility of topological behaviour?)

2) There could be more details added about the post-process correction of the resonators (lines 247-251). In particular, the range of additional pulses added would give readers a better sense of the consistency and reproducibility of the fabrication.

3) In the discussion of the interface between the SSH2 and SSH4 interface, in lines 286-288, it is mentioned that "The overall lattice can be considered either as an 11-site lattice attached to a 10-site SSH4 lattice, or a 10 site SSH2 lattice attached to an 11-site SSH4 lattice since the allocation of the shared site at the center is ambiguous". Why was this choice made? It should have been possible to choose a geometry with couplings where the interface can be clearly defined (although this may be wrong). Is this is not wrong, what motivated the authors to choose that particular configuration with ambiguity?

In conclusion, the manuscript deserves publication in Nature Communications, subject to optional minor revisions on the issues mentioned above.

Reviewer #5

(Remarks to the Author)
I co-reviewed this manuscript with one of the reviewers who provided the listed reports. This is part of the Nature Communications initiative to facilitate training in peer review and to provide appropriate recognition for Early Career Researchers who co-review manuscripts.

Version 1:

Reviewer comments:

Reviewer #1

(Remarks to the Author)
I am satisfied with the revised version of the manuscript. The authors have adequately addressed the comments and implemented the required corrections and changes. In my opinion, the manuscript is now suitable for publication in its present form.

Reviewer #2

(Remarks to the Author)
The authors have fully addressed my comments and I am happy to recommend the publication.

Reviewer #3

(Remarks to the Author)
I thank the authors for such a detailed response to my comments. The answers to the questions I posed are quite convincing; the edits made to the conclusion of the article regarding the prospects for further research on this system, in my opinion, have clarified the significance of the work. The manuscript does not contain any fundamental errors and, in its current form, is suitable for publication in Nature Communications.

Reviewer #4

(Remarks to the Author)
We believe the authors have addressed all the issues we raised, and the manuscript as resubmitted is acceptable for publication.

Reviewer #5

(Remarks to the Author)
I co-reviewed this manuscript with one of the reviewers who provided the listed reports. This is part of the Nature Communications initiative to facilitate training in peer review and to provide appropriate recognition for Early Career Researchers who co-review manuscripts.

Open Access This Peer Review File is licensed under a Creative Commons Attribution 4.0 International License, which permits use, sharing, adaptation, distribution and reproduction in any medium or format, as long as you give appropriate credit to the original author(s) and the source, provide a link to the Creative Commons license, and indicate if changes were made.
In cases where reviewers are anonymous, credit should be given to 'Anonymous Referee' and the source.
The images or other third party material in this Peer Review File are included in the article's Creative Commons license, unless indicated otherwise in a credit line to the material. If material is not included in the article's Creative Commons license and your intended use is not permitted by statutory regulation or exceeds the permitted use, you will need to obtain permission directly from the copyright holder.
To view a copy of this license, visit https://creativecommons.org/licenses/by/4.0/

April 20, 2026

We thank the editor for handling our manuscript "Multiband Topological Heterojunctions on the Surface Nanoscale Axial Photonics Platform" along with the Reviewers for their comments and insight. Below is a point-by-point response to their reviews, where we address the comments and concerns raised by the Reviewers and indicate how we have incorporated them into our revised manuscript. Note that in our responses to the Reviewer comments, when specific line numbers are mentioned, it is reference to the line numbers within the *marked-up redlined* version of the updated manuscript. Additionally, blue text refers to sections that have been added to the manuscript.

Reviewer #1 (Remarks to the Author):

This manuscript reports an impressive and timely experimental demonstration of multiband topological heterojunctions implemented on the SNAP platform, combining extraordinary fabrication precision with elegant topological photonics concepts. The work convincingly establishes SNAP as a uniquely powerful platform for analogue Hamiltonian simulation, especially in regimes that require sub-angstrom resonance matching across large resonator arrays, which is extremely challenging for integrated photonics.
Overall, the manuscript is well written, and of clear interest to the broad communities working on topological photonics, analogue simulation, and microresonator physics. The fabrication precision, spectral resolution, and level of experimental control are outstanding. I believe this work is very well suited for publication in Nature Communications after minor revisions.

We thank the Reviewer for their positive and detailed assessment of our work, recognizing the benefits of the SNAP platform for Hamiltonian simulation.

Comments 1.The statement that this approach is a "reversal of the tight-binding approximation" is interesting but could be made clearer. A short intuitive sentence explaining this statement would improve accessibility.

We agree. The sentence in question on lines 194-195 has been extended to:

"This methodology – utilizing the bound modes of a complicated physical system to approximate idealized sites on a lattice – can be considered a reversal of the widely used tight-binding approximation as a discrete lattice is approximated by the bound states of a physical system, as opposed to modeling the states of a physical system with a discrete lattice."

2. When reporting coupling strengths as wavelength splittings of several picometers, it may be helpful to translate these into frequency units (GHz) at least once, to give readers a better physical sense of the interaction scale.

Good point. An amendment has been made in our updated manuscript to the sentence on lines 296 to 297:

"Based upon the locations of the resonances, we estimate that the couplings of the fifth modal order are approximately $v_2 \approx 12$ pm and $v_1 \approx 36$ pm (equivalent to 1.5 and 4.5 GHz respectively), which are comparable to the predictions from Fig. 2 of $v_2 = 8$ pm and $v_1 = 30$ pm."

3. A definition of quasi-modes when they are first mentioned would improve readability, especially for non-experts in Green's-function spectroscopy.

We agree, and have updated our description of quasi-modes on lines 332 to 334:

"Because of this, two quasi-modes — lossy resonances with an energy spread (wavelengths) as opposed to a single energy — appear within the auxiliary bandgaps. These quasi-modes are artifacts of the SSH4 lattice's localized auxiliary bandgap modes after having been coupled to the SSH2 lattice."

4. A short intuitive explanation that band touching prevents assigning a unique bulk invariant would help readers connect this to standard topological phase-transition concepts.

We have incorporated this with the following additions to the description of the phase transitions (lines 434-435):

"The first method is to guess and check topology-preserving lattice deformations (those that do not cause the merger, splitting, or crossing of bands [1] and can thus be performed adiabatically) to manually search for the minimal number of interface modes. "

and then later adding the sentence (lines 448-451):

"However, the bands of the system can not be uniquely reconsidered as multiple conjoined bands. Additionally, this puts the system directly between two topological phases (a topological critical point), making eq. 7 ill-defined."

5. The CO2 laser beam drawn in Fig. 1(a) looks more like a parallel than a focused beam. Since the fabrication relies on strong spatial localization of heating, it would be helpful to redraw the beam with a visible waist at the fiber surface. As it stands, the image may mislead readers unfamiliar with the SNAP fabrication process.

We have adjusted the shape of the beam in Fig. 1(a) to better reflect the physical shape of the beam.

6. Occasional misprints appear, e.g., "except except" in Sec. 4 and "is is clear" in Appendix B.

We appreciate the Reviewer pointing these out and have fixed them.

Reviewer #2 (Remarks to the Author):

This manuscript presents a well-executed experimental and theoretical study on the realization of topological photonic lattices using the SNAP platform. The work demonstrates the experimental implementation of SSH models and their multiband heterojunctions in SNAP devices, supported by rigorous theoretical analysis. The results are novel, the methodology is sound, and the findings are of potential interest to the broader communities in topological photonics.

We thank the Reviewer for their assessment of our work.

1. While the manuscript cites relevant literature, a more explicit comparison with other platforms (e.g., integrated photonic circuits, superconducting qubits) in terms of performance metrics (Q-factor, scalability, tuning precision) would strengthen the case for SNAP's advantages.

Certain advantages and disadvantages of photonic integrated circuits (PICs) have been introduced into the paragraphs discussing photonic AHS and SNAP as a platform on lines 80, 86-88, and 96-97. Additionally, the scalability of SNAP is now expanded in our revised manuscript's discussion of simulating higher-dimensional lattices in the Conclusion (lines 509-521).

Explaining the advantages of non-photonic AHS over photonic AHS is beyond the scope of this paper, which is why such metrics have not been included for other platforms such as superconducting qubits - photonic AHS is already established [2] and we are proposing an alternative to its previously reported implementations in the literature. Summarizing in brief here, the advantages of photonics over superconducting circuits come from its room-temperature ambient operation, compactness and scalability due to much smaller wavelengths [3]. Advantages of SNAP within photonics platforms, as the Reviewer notes, indeed result from the high Q-factor, ease of fabrication and post-fabrication non-volatile tuning, and precision of tuning as opposed to integrated photonic chips and other platforms. There are of course limitations that arise from the low nonlinear interaction strength between photons compared to superconducting qubits.

The claim that this is the "first heterojunction of higher-order SSH lattices" should be supported by a brief discussion of why such structures have not been realized elsewhere, if possible.

We have added several sentences discussing this in the Introduction (lines 108-117),

"Such interfaces have not been well-studied theoretically [4], which we address by generalizing topological polarization analysis methods to higher-order SSH lattices, revealing how "fake" topological interface modes contingent on the interface's implementation may emerge. The structure is composed of 21 frequency-matched adjacent SNAP microresonators with evanescent couplings controlled by the intersite spacings and dependent on which axial mode resonances within the SNAP resonator are utilized. We thus demonstrate SNAP's excellent suitability for investigation of Hamiltonians with interesting topological properties and conclude with discussion on prospects for expanding such investigations to two- or higher-dimensional topological models."

In short, the theory of SSH heterojunction topology is itself new and less explored, which explains why experimental realizations have not been realized elsewhere. Specifically for the SNAP platform, the precision fabrication required to create such frequency-matched yet nonuniformly coupled SNAP resonator arrays, is what we believe has hindered previous realizations of our demonstrated topological effects.

2. The manuscript briefly mentions nonlinear effects and synthetic dimensions as future directions but does not critically discuss the current limitations of the SNAP platform (e.g., maximum lattice size, sensitivity to environmental noise, scalability beyond 1D). A dedicated paragraph on challenges and prospects would provide a more balanced perspective.

We have expanded that discussion in the conclusion discussing the future possibilities of scaling the SNAP platform (lines 509-521). We address scalability beyond 1D directly therein. Environmental noise susceptibility is not currently a noteworthy limiter, and can be addressed with appropriate packaging. The maximum lattice size is not uniquely defined and involves complex interdependency with the chosen approach (from among many options) for giving rise to the target effective radius variation profile, placing such a discussion beyond the scope of this work.

3. The development of a generalized topological polarization method for multiband heterojunctions is a notable theoretical contribution. However, the connection between Eq. (6)-(7) and physical observables could be elaborated. A more intuitive explanation of the "sublattice displacement" and its relationship to measurable quantities would strengthen the theory section.

We have added lines describing how the topological phases upon which the sublattice displacements are defined could be measured (lines 406-409):

"Physically, the integral counts the winding of the wavefunction phase between the s_{L+1} sites

and s_0 sites as one circumnavigates a Brillouin zone, implying it could be measured through interferometric techniques and coherent detection."

Additionally, we have added an explanation of the meaning of sublattice displacement within the new appendix D, which is referenced on line 423:

"Furthermore, the sublattice displacements at either ends of the lattice can be directly related to the amount of bound states at that end of the lattice (Appendix D)."

Within Appendix D, we show that $Q_+(s_L)$, the number of bound states on the right side of a lattice whose last site is s_L, can be expressed as

$$Q_+(s_L) = [s_{L+1} - 1] + \left[d(s_{L+1}) - \bar{d}\right] + \Delta Q$$

where $d(s) = D(s) - s$ is the "relative" sublattice displacement, $\bar{d}$ is the average of all the relative sublattice displacements within the sublattice (effectively setting the gauge of d), and ΔQ is an additional topological invariant quantifying the inversion asymmetry of the lattice (defined in Appendix D). A similar equation also exists for the number of bound states on the left side of the lattice, $Q_-(s_1)$.

4. The extension of the method to higher dimensions is suggested but not elaborated. A brief speculative discussion on how SNAP could emulate 2D/3D topological models would highlight the platform's future potential.

We appreciate this helpful feedback and have expanded upon how extra dimension for the SNAP resonator lattices could be formed in a new paragraph at the end of the conclusion (lines 509-521):

"While this work is focused on 1D Hamiltonians, it serves a stepping stone towards implementing two- and higher-dimensional topological lattices. SNAP fibers' resonances' sensitivity to radii make frequency- and dispersion-matching distinct fibers difficult, affecting the scalability in coupling numerous SNAP devices by either evanescently coupling adjacent fibers [5, 6] or by using additional tapered fibers as interconnects. However, the small mode volume and low losses of SNAP resonators enable nonlinear effects to manifest at low intensities [7, 8], opening the possibility for both the axial quantum numbers and the non-axial quantum numbers to be utilized as a synthetic dimension [9]. Specifically, different frequency modes (due to either a difference in axial, azimuthal, radial, or polarization quantum numbers) can be coupled together through Kerr-induced four-wave-mixing[10] by exciting the resonator with auxiliary lasers that satisfy the requisite energy and momentum conservation conditions."

5. The authors discussed various Hamiltonian simulation platforms; however, many of the cited references are rather dated. It is recommended that the authors include more recent publications to better reflect the current progress in the field. For instance, Science Bulletin Volume 70, Issue 8, 30 April 2025, Pages 1347-1360; Nature Reviews Physics, Volume 7, pages 606–620 (2025); Photonics Insights, Vol. 4, Issue 2, R06 (June 2025).

We thank the Reviewer for pointing out the more recent references. The works the Reviewer noted have been incorporated as references [9] and [17-18] in the manuscript.

Reviewer #3 (Remarks to the Author):

This manuscript investigates the structure of axial modes in a system of coupled SNAP microresonators. Using a well-known technique of introducing an effective radius variation into a standard optical fiber by heating it with a CO2 laser (10.1364/OE.20.027896, 10.1364/OE.20.010684), a system of coupled resonators

was implemented that forms the Su-Schrieffer-Heeger (SSH) lattice. The approximation of the measured transmission spectra of the fabricated system was performed using the theory developed by Mikhail Sumetsky (10.1364/OE.19.026470, 10.1364/OE.20.022537), in analogy with how this was done for other systems of coupled SNAP resonators (10.1364/oe.21.015268, 10.1364/ol.433077).

We thank the Reviewer for their assessment of our work.

The results of the work show the presence in such a system of both edge topological modes and axial modes arising at the junction of changes in the lattice period. The authors argue that the SNAP platform is the most suitable for analog Hamiltonian simulation, which is undeniable, because the axial part of the wave equation, which describes the field distributions in a cylindrical resonator with a radius variation, coincides in its form with the one-dimensional stationary Schrödinger equation. This also opens the possibility to study topological modes in 1D lattices.

We are glad that the Reviewer is convinced about the SNAP platform's suitability for analogue Hamiltonian simulation.

However, the SNAP axial modes have a critical distinction from those in other topological photonic systems. In various types of topological photonic systems, the study of which has gained popularity recently, the presence of topological modes in the system leads to the emergence of non-trivial linear and nonlinear dynamics (10.1038/s41467-018-05461-5, 10.1038/s41567-021-01492-w, 10.1038/s41467-025-59941-6, 10.1038/NPHOTON.2014.248, 10.1038/s41467-023-40238-5, 10.1038/s41566-017-0048-5, 10.1038/nphoton.2013.274). In the SNAP system, the axial modes are stationary along the microresonator axis, and the field dynamics lie in the perpendicular plane. This makes it unclear what non-trivial dynamics can be expected in this system. Topological axial modes and axial modes numbered q=1-3 (Fig. 1) are equally isolated from other axial modes, and from the perspective of the linear and nonlinear dynamics that can develop in the fiber cross-section, it seems that it makes no difference which mode we pump with the radiation source. In both cases, we will have a stationary field along the microresonator axis, and the topological property of the axial distribution, due to the smallness of the effective radius variation, cannot affect the spatial distribution and dynamics of the field in the cross-section (this is the fundamental assumption of the theory when we proceed to separate the variables of the wave equation).

We respectfully disagree with key aspects of this assessment. The Reviewer correctly points out that the radial and angular distribution of light remains effectively unchanged so long as a single frequency mode is being stimulated, as associated with the separation of variables in the theory. However, there is nothing special about the azimuthal and radial degrees of freedom for the purposes of investigating optical systems with non-trivial topology and associated interesting behaviors. The topological behavior we demonstrate herein involves a single dimension which we specify as the the longitudinal dimension of the SNAP fibers that host a 1D array of perturbation-induced-microresonators.

The SSH model with which we explore topological photonics on the SNAP platform is an important and well-established prototypical model of 1 dimensional topological behavior [1] – it has long been possible to produce topological behavior with just a single dimension [11]. The second DOI cited by the Reviewer in their references to interesting dynamics, (doi.org/10.1038/s41567-021-01492-w, Nature Physics 2022), demonstrated as a main claim the observation of SSH edge modes, and was co-authored by the last author on the current manuscript. A quintessential topological property of 1D topological lattices is the existence of intra-bandgap modes localized to the boundaries. Producing those topological edge states (clearly displayed in Fig. 1d, 3b

and 4e) and verifying that they abide by the new generalized SSH heterojunction theory we developed are two key results of our paper, opening the door to a plethora of other kinds of topology. For example, by modulating the inter-resonator couplings and on-site couplings at a speed comparable to the photon lifetime (e.g., through nonlinear Kerr effects induced by additional lasers), the photons could be topologically pumped along the axial dimension [1]. Additionally, by intentionally creating loss-inducing defects like in Fig. 1(c), non-Hermitian 1D topological phenomena arising near exceptional points through variations in the photon lifetime between the sites system could be explored [11]. In terms of nonlinear dynamics, the topological localization of the bound modes within SSH lattices has been utilized to induce lasing [12] and enhance third-harmonic generation [13], which are realistic in our SNAP system due to its high-Q-induced large resonant enhancement. These are just some examples of nontrivial linear and nonlinear dynamics within the reach of our system, albeit through arduous future experiments.

Furthermore, the light is not truly stationary within the individual resonators (except approximately so in the well-isolated lower-order axial modes) due to the inter-resonator couplings: light coupled into any part of the potential well will be able to tunnel between resonators and propagate along the axial dimension throughout the entire potential well of the device. This was well demonstrated in [14] where they showed how chains of coupled SNAP microresonators can serve as optical delay lines whose delay is proportional to the length of the resonator chain, indicating propagation of light pulses throughout the entire structure (Fig. E1). Additionally, it has been explained how SNAP device can be used in a four-port (add/drop) configuration where photons are coupled into SNAP device at one location (added), and coupled out at another location (dropped) [15]. Towards maximal clarity, we illustrate a notional 3D light path through a system described by a 1D potential in Fig. E1(d).

[REDACTED]

Fig. E1 (a-c) Experimental data from Ref. [14] demonstrating how a multi-resonator SNAP device can be utilized as a delay line with a duration proportional to the number of coupled resonators. The experimental setup is depicted in **(a)**, and the measured **(b)** and theoretical **(c)** intensity of incident and transmitted light. In both cases, there is a fixed duration of time between pulses indicating the time necessary for photons to propagate to the end of the lattice and back. **(d)** Diagram indicating one notional path of light through a SNAP device in a four-port configuration[15]. Both the the data in (b,c) and the schematic (d) establish that the SNAP axial modes are not stationary along the microresonator axis, but instead follow nontrivial and sometimes complicated pathways within each resonator and across multiple coupled resonators, contrary to Reviewer #3's statement.

For example, in systems of coupled ring microresonators, nontrivial dynamics and topological soliton solutions are observed because the radiation propagates in the plane of topological modes (10.1126/science.ado0053, 10.1038/s42005-023-01438-z). A SNAP microresonator is analogous to a system of coupled ring microresonators arranged in a row and placed edgewise. Clearly, in this case, the field

dynamics along the perimeter of the rings will coincide with the dynamics in a single ring.

While the Reviewer is correct that a SNAP microresonator is analogous to an array of coupled microrings placed edgewise, and the axial modes are analogous to the coupled-ring-array's supermodes (hybridized eigenmodes), the focus of our manuscript is not to compare the dynamics in this edgewise array of rings with the dynamics in a single ring. To compare with the "system of coupled ring microresonators" whereby the "radiation propagates in the plane of topological modes," one should look at our *array* of coupled SNAP microresonators rather than a single SNAP site whose single axial mode dynamics are comparable to a single ring's perimeter dynamics. In this more suitable analogy, the dynamics in a planar array of single-mode rings can be compared with the dynamics of supermodes arising from hybridization of modes with common q in an array of SNAP microresonator sites. An added advantage of SNAP systems is that multiple such lattices with different coupling coefficients can be simultaneously realized in different axial mode families. The Reviewer's concern that nontrivial topology/solitons are difficult to observe in our SNAP array was already answered in our previous response, but briefly, the 1D lattices we show also have nontrivial topological solutions and we have added explanations (lines 509-521) of how our system could be extended to explore 2D dynamics by coupling axial modes and harnessing synthetic dimension ideas.

Furthermore, the claim that the theory can be generalized to a two-dimensional lattice also seems non-obvious and was not explained in the text of the article. The linear coupling between modes with different polarizations and radial numbers is not experimentally controlled and arises due to Rayleigh scattering by fiber defects. For such coupling to be effective, the resonant wavelengths of axial series with different radial numbers or with different polarizations must overlap. Such overlap is possible, but it will lead to a linear coupling between modes with different axial numbers in the series. It is not entirely clear how such a coupling can be used for scaling.

For experimentally realizing 2D lattices, it is not our intention to rely upon Rayleigh scattering to couple modes with distinct polarization, angular, or radial quantum numbers; the Reviewer indeed correctly notes the reasons (lack of good control) that would make it impractical. As described in [16], coupling between resonant modes at different frequencies can be achieved through nonlinear optical processes such as four-wave-mixing, which has already been demonstrated within SNAP resonators [8]. Additionally, linear coupling between adjacent SNAP fibers would be possible, though arduous.

It is helpful feedback that the path towards generalization to 2D lattices was insufficiently clear in our originally submitted manuscript. To address this concern, which was also raised in Reviewer #1's comment 4, we have better elaborated on future possibilities for higher dimensions in a new paragraph in the Conclusion section (lines 509-521) that is detailed in the response to Reviewer 2.

Though the present work demonstrates a new configuration of the SNAP platform with topological properties of axial modes, it cannot make a broad impact because non-trivial light dynamics are associated with azimuthal modes only. Therefore, I recommend publishing this manuscript elsewhere in a more specialized journal.

Through revisions to our manuscript and discussions in this response, we have endeavored to clarify these points and emphasize that our approach supports investigation of nontrivial topological behavior as we have demonstrated in 1D with both SSH-2 edge modes and in a novel heterojunction (SSH-2/SSH-4), and in our expanded Conclusion (lines 509-521) explaining future opportunities to implement nontrivial topological dynamics beyond 1D lattices. This includes experimentally feasible techniques e.g., four-wave mixing nonlinearities for frequency

synthetic dimensions and linear-optical coupling between SNAP fibers to create lattices in the spatial dimension. We note that a demonstration of nontrivial topology, even in 1D, is in itself quite significant for a new topological photonics platform such as SNAP: Reviewer #4 wrote, "there are no other topological systems implemented in SNAP resonators." We already go beyond a mere demonstration of topology (SSH-2) by extending to heterojunctions between dissimilar SSH-2 and SSH-4 lattices. Taking all this into account, we hope to convince the Reviewer about the broad impact of our results, making it suitable for Nature Communications.

Reviewer #4 (Remarks to the Author):

The authors report the implementation of an optical analog of a Hamiltonian system: the Su-Schrieffer-Heeger (SSH2) model and one generalization (SSH4). The experimental implementation is made with surface nanoscale axial photonics (SNAP) optical microresonators, of a simple 5-unit SSH2 chain, and a longer chain with an interface between SSH2 and SSH4 lattices. The authors then introduce theory to explain what happens at the interface of an SSH2 and SSH4, since that topic has been less explored. Overall the manuscript is well written, very clear, and all the claims are supported with credible evidence. The topic is very timely, as optical implementations of topological systems are being actively explored and, as far to our current knowledge, there are no other topological systems implemented in SNAP resonators.

We thank the Reviewer for their assessment of our work, recognizing that topological photonics has not been previously implemented in SNAP resonators.

The analysis of the coupling as a function of the distance between resonators was particularly interesting.

We are happy to hear this.

There no major issues in the manuscript to point out, but rather a set of minor points that could potentially be clarified. These are mostly in the experimental part, due to a lack of theoretical depth to find out serious issues in the theoretical discussion (which looks fine to a lightly trained eye).

We are glad the Reviewer found no major issues and endeavor to address the minor points below.

1) According to the fabrication description, the post-processing was focused on optimizing the q=3 axial mode lattice (lines 312-313). Then, the analysis is centered on the axial mode lattices with q=4,5 (with good reason, as those show very clear effects). When looking at Figure 3a, it seems that the q=3 mode does have some hint of coupling showing some splitting of states. A bit of analysis on what is going on in that case would be a good addition (ie are the couplings low enough that there is no possibility of topological behaviour?)

Regarding the comment on a hint of coupling showing some splitting in the $q = 3$ modes, the reason is twofold: (1) Residual mismatch between the SNAP resonator sites that makes the $q = 3$ axial mode frequencies vary from site to site, despite post-fabrication corrections, and (2) A small but nonzero coupling between adjacent sites in this axial mode family, as evident from Fig. 2. However, this coupling-induced splitting for identical resonators is barely resolved, as indicated in Fig. 2(a), and hence the primary contribution is from the first reason of residual post-fabrication mismatch. We have added a sentence in lines 280-283 regarding this:

"There is some mode coupling apparent between the $q = 3$ modes, but the gap between the

modes arises primarily from the residual resonator mismatch, thus we omit analysis of the $q = 3$ mode."

Additionally, a sentence has been added in lines 299-304 to elaborate upon why the other lattices are neglected:

"We can also infer that the decay lengths for $q < 3$ are much less than 1 unit cell (implying the edge states are effectively just position eigenstates), and the decay lengths for $q > 6$ are more than 5 unit cells (implying the edge states diffuse across the entire bulk), explaining why those modes do not noticeably drop in intensity as they leak into the bulk."

2) There could be more details added about the post-process correction of the resonators (lines 247-251). In particular, the range of additional pulses added would give readers a better sense of the consistency and reproducibility of the fabrication.

This is a good point. We added discussion of the number of post-processing rounds on lines 267-268:

"This process was repeated about 10 times until the resonances of the relevant axial mode order(s) of the microresonators were matched to within desired tolerance. "

3) In the discussion of the interface between the SSH2 and SSH4 interface, in lines 286-288, it is mentioned that "The overall lattice can be considered either as an 11-site lattice attached to a 10-site SSH4 lattice, or a 10 site SSH2 lattice attached to an 11-site SSH4 lattice since the allocation of the shared site at the center is ambiguous". Why was this choice made? It should have been possible to choose a geometry with couplings where the interface can be clearly defined (although this may be wrong). Is this is not wrong, what motivated the authors to choose that particular configuration with ambiguity?

The reasoning in picking the SSH2/SSH4 interface that way was that we wanted to show a variety of localized interface modes. The ambiguity was accidental, not intentional as the presented experimental work preceded the theoretical work, and addressing the ambiguities in classifying the heterojunction of Fig. 4 was actually the impetus for developing the new classification and analysis methods. We would like to clarify that the ambiguity does not undermine any of our results, since the irrelevance of the exact implementation of an interface to its topological properties is a central claim of our paper and is in line with the tenet of topological physics that local properties and disorder do not affect topological properties.

Regardless, ambiguities in these systems can not be avoided without intentionally introducing on-site couplings (resulting in a Rice-Mele lattice [1]), which breaks symmetries and obscures topological effects in SSH-like systems. For example, expressing the coupling between sites j and $j+1$ as the function $v(j)$ which obeys $v(j) = v_L(j)$ for $j \leq j_I$ but $v(j) = v_R(j)$ for $j_I < j$, then the site j_I can be considered part of either the left or right lattices since its coupling to the left $v(j_I - 1) = v_L(j_I - 1)$ and its coupling to the right $v(j_I) = v_R(j_I)$ follow the their sides' pattern respectively. To avoid the ambiguity would require making the coupling between the two lattices uncharacteristic of either sides' pattern (i.e. $v(j_I) \neq v_L(j_I)$ and $v(j_I) \neq v_R(j_I)$). In that situation, site j_I could only be attributed to the left side, and site $j_I + 1$ could only be attributed to the right side. However, that introduces the ambiguity of what the value of $v(j_I)$ would be if it is neither $v_L(j_I)$ or $v_R(j_I)$. The ambiguity come from the fact that we impose a pattern on the couplings between sites, not on the sites themselves. To address this question for the readers, we have added an aside to lines 319-320:

"(such ambiguity of the sites can not be avoided without imposing distinct on-site potentials/self-couplings patterns for both lattices)"

In conclusion, the manuscript deserves publication in Nature Communications, subject to optional minor revisions on the issues mentioned above.

Reviewer #5 (Remarks to the Author):

I co-reviewed this manuscript with one of the reviewers who provided the listed reports. This is part of the Nature Communications initiative to facilitate training in peer review and to provide appropriate recognition for Early Career Researchers who co-review manuscripts.

We thank the Reviewer for their assessment of our work and hope they found the experience valuable.

---

*References

1. J. Asboth, e. a. *A Short Course on Topological Insulators: Band Structure and Edge States in One and Two Dimensions* 1st ed. 2016. **919** (Springer International Publishing, 2016).
2. Aspuru-Guzik, A. & Walther, P. Photonic quantum simulators. *Nature Physics* **8,** 285–291 (2012).
3. Georgescu, I. M., Ashhab, S. & Nori, F. Quantum simulation. *Reviews of Modern Physics* **86,** 153–185 (2014).
4. Zhao, T. *et al.* Interface states and optical coupling functionalities in the super-SSH lattices. *Optics Express* **32,** 21487–21496 (2024).
5. Sharma, I. & Sumetsky, M. *Widely tunable SNAP microresonators via translation of side-coupled optical fibers* 2026.
6. Sharma, I. & Sumetsky, M. Widely FSR tunable high Q-factor microresonators formed at the intersection of straight optical fibers. *Optica* **12,** 890–893 (2025).
7. Pöllinger, M. & Rauschenbeutel, A. All-optical signal processing at ultra-low powers in bottle microresonators using the Kerr effect. *Optics Express* **18,** 17764 (2010).
8. Eadie, E., Deep, S. & Bianucci, P. Azimuthal Kerr combs with predictable mode spacing in surface nanoscale axial photonic microresonators. *Optics Express* **33,** 34677–34687 (2025).
9. Yu, D. *et al.* Comprehensive review on developments of synthetic dimensions. *Photonics Insights* **4,** R06 (2025).
10. Tusnin, A., Tikan, A., Komagata, K. & Kippenberg, T. J. Nonlinear dynamics and Kerr frequency comb formation in lattices of coupled microresonators. *Communications Physics* **6,** 317 (2023).
11. Hotte-Kilburn, A. & Bianucci, P. Integrated topological photonics in one dimension. *Advances in Physics: X* **10,** 2476417 (2025).
12. Parto, M. *et al.* Edge-Mode Lasing in 1D Topological Active Arrays. *Physical Review Letters* **120,** 113901 (2018).
13. Kruk, S. *et al.* Nonlinear light generation in topological nanostructures. *Nature Nanotechnology* **14,** 126–130 (2019).
14. Sumetsky, M. A SNAP coupled microresonator delay line. *Optics Express* **21,** 15268–15279 (2013).
15. Crespo-Ballesteros, M., Yang, Y., Toropov, N. & Sumetsky, M. Four-port SNAP microresonator device. *Optics Letters* **44,** 3498 (2019).

16. Kolesnikova, A. Y. & Vatnik, I. D. Theory of nonlinear whispering-gallery-mode dynamics in surface nanoscale axial photonics microresonators. *Physical Review A* **108,** 033506 (2023).
